# Current Understanding of the Effects of Congestion on Traffic Accidents

**DOI:** 10.3390/ijerph16183400

**Published:** 2019-09-13

**Authors:** Angus Eugene Retallack, Bertram Ostendorf

**Affiliations:** Faculty of Sciences, School of Biological Sciences, The University of Adelaide, North Terrace Campus, Adelaide, SA 5005, Australia; angus.retallack@adelaide.edu.au

**Keywords:** traffic accidents, congestion, traffic volume, real-time traffic data, Bluetooth

## Abstract

Traffic accidents impart both economic and social costs upon communities around the world, hence the desire for accident rates to be reduced. For this reduction to occur, the factors influencing the occurrence of accidents must be understood. The role of congestion in modifying accident risk has been widely studied, but consensus has not been reached, with conflicting results leaving open questions. An inverse relationship between accidents and congestion would imply a benefit of congested conditions for road safety, posing a difficult situation for traffic management. This paper assesses articles that reveal the shape of the relationship between traffic accidents and congestion. We find a positive linear response to dominate the literature. However, studies with higher numbers of statistical units tend to show a U-shaped relationship. This suggests an important role of high spatio-temporal traffic data in understanding factors causing accidents and identifying the combination of real-time conditions which may lead to increased accident risk. Modern advancements in traffic measurement systems provide the ability for real-time alleviation of accident-prone conditions before they can fully develop.

## 1. Introduction

In 2014, road accidents were estimated to cost Australia AUD $27 billion per year [1]. However, traffic accidents can also have ‘social costs’—costs which are imposed upon others, such as travel time delay and emotional suffering, in the event of an accident [2]. In the United States, preretirement deaths resulting from traffic accidents outweigh the toll taken by the two most deadly diseases, cancer and heart disease, while nearly half of the deaths of 19-year-olds can be attributed to traffic accidents [3]. In Australia, annual road deaths per 100,000 people decreased from 26.6 to 5.1 between 1975 and 2013, placing Australia 16th of 33 countries with this data available [1]. Although, while fatal accidents are declining, there was over 1000 road fatalities in 2014 [1]. To continue this declining trend, the wide range of factors influencing the occurrence of accidents must be understood. These factors include traffic and road infrastructure characteristics, environmental conditions, vehicle design and human factors [4]. From 1990 to 2006, driver error, distraction or unintended impairment (i.e., sun in eyes) were recorded as being the most common major factors in fatal accidents in Australia [5].

Congestion is an interesting variable which can influence the likelihood of a traffic accident. While it may be assumed that congestion should be reduced, if its correlation to fatal and serious injury accidents is negative then the presence of congested conditions would actually be beneficial to road safety, posing a difficult situation for traffic management [6]. To solve this conundrum, and work towards reducing accident rates, it is important to gain a strong understanding of the effects of congestion on traffic accidents. This is an area which has been studied for close to 100 years [7] with mixed results. Positive linear relationships between traffic volume and total accidents are a common finding [8,9,10,11], as are U-shaped functions, where accident occurrence is greatest in low and high levels of congestion [12,13,14,15,16]. Shefer [17] proposed that, when considering only fatal accidents, the opposite would be observed; fatalities were predicted to be greatest at median levels of congestion and lowest when congestion was low and high.

Increased understanding of the causes of traffic accidents, particularly congestion, would go towards reducing the economic and social costs associated with accidents. This could be achieved through the facilitation of improved traffic infrastructure design and traffic management. Modern advancements in traffic measurement systems could also serve to reduce accidents, with the potential to identify hazardous conditions in real-time, allowing action to be taken to avoid increased accident risk [18]. Of particular interest is the use of real-time traffic measurement technologies, which can identify unsafe traffic conditions as they are developing, allowing measures to be taken to alleviate these conditions [19]. Bluetooth traffic sensors are one method used to collect this high temporal resolution traffic data which can be used for measuring crash risk, such as in studies by Yuan, et al., [20], Yuan, et al., [21], and Yuan and Abdel-Aty [22]. Studies investigating the effects of traffic and weather on traffic accidents are generally focused on freeways and urban expressways, with urban areas being less examined [23]. While sources of real-time traffic data such as remote traffic microwave sensors (RTMS) and automated vehicle identification (AVI) systems are often available on highway systems, Bluetooth sensors can be used to measure traffic conditions in urban locations [20]. This highlights Bluetooth technology as a possible method for filling the gap in urban studies. Although, in Yuan’s studies in Florida [20,21,22], Bluetooth detectors were used for collecting travel speed data, while 15-min traffic volume data was collected using adaptive signal controllers. This poses the question as to whether it would be viable to use Bluetooth detectors as the sole source of traffic data, using travel time as a proxy for levels of congestion; a potential direction for future development and use of this new technology. Applying data from these sensors on a larger scale would also be an interesting direction, with the studies in Florida [20,21,22] being confined to 23 intersections.

In light of these recent developments in traffic measurement technology, it seems appropriate to review the results of past studies; looking into the causative factors, specifically congestion, which influences the occurrence of traffic accidents. Of additional focus is the how the characteristics of data used can influence results, and if the conclusions of earlier studies may have been limited by the use of aggregated spatio-temporal traffic data. A summary table relating the spatio-temporal resolutions of traffic data to the results achieved will seek to unveil any pattern between higher quality data and more detailed results with the purpose of directing future choices of traffic data sources.

## 2. Factors Influencing Accidents

The mixed results of studies investigating the effects of congestion on traffic accidents can be at least partially attributed to the extremely large number of contributing factors, some of which may act in combination with each other [7]. Understanding what these factors are and how they influence the likelihood of accidents is necessary in taking steps to reduce accident rates.

There is strong evidence that environmental conditions, such as rain or light levels, affect the frequency of accident occurrences. In Montreal, Canada, increased snow and rain related to increased numbers of road accidents [24], with other studies noting the effects of rain often becoming stronger with increased time since the last rainfall event [25,26]. The number of accidents increased with increasing rainfall in Southern Illinois, where accident severity increased in some cities and decreased in others, highlighting the complexity of the factors influencing traffic accidents [27]. Veh [7] found that nearly half of the accidents on New Jersey highways occurred at night, despite these hours only accounting for one fifth of total daily traffic. On rural highways in Connecticut, single-vehicle accidents occurred most often in the evening and night [28]; these accidents could be a result of increased drowsiness [28] or decreased visibility [29].

A human element to accident occurrence also exists and relates to driver characteristics such as age, behavior, alcohol consumption, and distraction by passengers [30,31]. Drivers who were determined to be involved in risk-taking behavior, based on a questionnaire, were more often involved in near-miss accidents and accidents causing injury or property damage [32]. People who frequently engaged in drink driving had accident rates 2.6 times higher than those who did not [33].

Vehicle factors contribute to the severity of accidents in a number of ways. Fatality risks are 13 times greater in a 900 kg car than in an 1800 kg car if they were to collide and 2.4 times greater when comparing single vehicle crashes [3]. Modern safety features such as autonomous emergency braking can avoid crashes and reduce their severity by lowering collision speeds. This feature is expected to prevent over 1200 fatalities and 54,000 hospitalized injuries in Australia by 2033 [1]. Electronic stability control caused reductions of over 25% in serious injury or fatal accidents occurring in Sweden, increasing to around a 50% reduction in low traction conditions [34].

The effects of road design on accidents can be seen in Washington, where collisions increased as the number of curves on a segment of road increased [35] and sharp curves and narrow lanes were found to decrease accident frequency [36].

Congestion can also affect the occurrence of traffic accidents, although not necessarily in a way which would at first seem logical. While accidents may be expected to increase due to a larger number of vehicles on the road, they may also decrease due to a reduction in speed. The latter was found in the US, Israel, and Germany when only fatal accidents were considered [29]. In Michigan the highest accident rates were observed at the lowest and highest volume to capacity (v/c) ratios [15]; this suggests that accident occurrence may be a function of both speed and number of cars on the road. That is, at low traffic volumes accidents occur frequently as a result of high speeds, while at high volumes, accident rates are higher due to more cars being on the road. Reducing congestion is beneficial in reducing delays, pollution, and stress and increasing productivity and its associated economic benefits [37]. However, the increase of fatal accidents at low levels of congestion [14] creates a conflicting scenario where congestion and fatalities cannot be reduced together. This emphasizes the importance of improving the understanding of the influences of congestion on accidents.

## 3. Effects of Congestion on Traffic Accidents

Congestion as a factor influencing traffic accident rates has been studied for many years. One of the earliest studies was conducted by Veh [7] who determined that as ADT increases, so does the number of accidents per million vehicle miles—up to an average daily traffic of 7000 vehicles. After this there is a gradual decrease in accident rates, likely as a result of increased congestion resulting in decreased speed, a trend also found by Raff [8]. Woo [10] found a positive correlation between accidents and both AADT and congestion index (AADT/road capacity). Similar correlations were also found in Oregon [9,11], Romania [38], and New York [39], with increased accident rates being attributed to increased traffic volumes. Also using AADT, in three US states, this same positive correlation was found for multi-vehicle accidents. However, when single-vehicle accidents were considered, the relationship was the opposite and accident rates declined as traffic volume increased [40].

While the correlations found by Woo [10], Schoppert [9], and Head [11] were deemed strong enough to enable AADT or ADT to be used to predict accident rates, the use of higher temporal resolution hourly traffic volumes would likely give a stronger relationship [12]. Using this improved traffic data, a U-shaped curve was seen between hourly traffic volume and accident rates; the highest rates occurred when traffic volume was either low or high, with the lowest rates occurring at traffic volumes of around 1500 vehicles per hour [12]. In Israel the relationship between hourly traffic volume and total accidents also produced a U-shaped curve [13]. While the difference in this relationship when compared to other studies which presented simple linear correlations [8,9,10,11] could be due to differences between study locations, the higher temporal resolution of the hourly data may serve to uncover finer detail in the relationship between the two factors. This U-shaped relationship may also be a combination of the single and multi-vehicle accident correlations seen by Kihlberg and Tharp [40]—the high accident rates at low levels of congestion could be attributed to single-vehicle accidents, while the accidents at high levels of congestion may be due to multi-vehicle accidents.

Level of service (LOS) is another important concept in traffic management. LOS is a method initially proposed in the 1965 Transportation Research Board Highway Capacity Manual [41] and describes the operating conditions of traffic and how they are perceived by motorists [42]. Conditions such as speed, travel time, and freedom to maneuver are considered, with road segments being grouped into one of six levels—ranging from A (best) to F (worst) based on these conditions [43]. The maximum flow rate which can be achieved at each LOS can then be established by calculating the ‘service flow rate’. This is defined as the maximum hourly rate at which vehicles can be reasonably expected to drive a segment of road while still maintaining the conditions of that LOS [43].

While Dart and Mann [44] averaged traffic volume data over 5 years for each road section, straying from the idea that hourly traffic volume may provide stronger results [12], they used a traffic volume ratio rather than the more basic ADT to quantify traffic volume. This ratio was calculated by dividing peak hourly traffic volume by the service flow rate for LOS B, as defined in the Highway Capacity Manual [45]. Similar results to studies which used AADT were found, with a simple positive correlation between the traffic volume ratio and accident rates being observed [44]. This further supports the idea that low temporal resolution traffic statistics struggle to uncover a second order term in the regression.

The volume to capacity (v/c) ratio is another method which can be used to measure congestion and is beneficial due to the different effects a volume of traffic will have on different road segments. While a certain volume of cars travelling down a narrow road (low capacity) may result in poor traffic conditions, the same volume travelling down a road with a greater capacity would have a smaller effect [14]. In Detroit, accident rates were highest when v/c ratios were lowest and decreased with increasing v/c ratio before increasing again as the v/c ratio continued to increase [15]. When investigating the effects of v/c ratio on accident rates, however, it is important to ensure the study location experiences a broad range of traffic volumes. In New Mexico, Hall and Pendleton [46] concluded that the highest accident rates occur at low v/c ratios, however, due to the road segments selected rarely exceeding a v/c ratio of 0.5, they were unable to gather sufficient data to determine the effects of high traffic volumes.

Both v/c ratios and LOS were used in a study in Greece. Accident rates remained relatively constant for LOS A-C up to a v/c ratio of 0.65 before nearly doubling at LOS D and increasing again at LOS F [14]. When only fatal accidents were considered, a U-shaped relationship was seen, similar to results by Ceder [13] and Gwynn [12]. The incremental nature of this relationship however, with accident rates being plotted against LOS, is a disadvantage of the LOS measurement of traffic congestion as a continuous range of values are not provided [47].

Contrary to the Greek results, Shefer [17] hypothesized that when considering only fatal accidents, a bell-shaped curve would result from the relationship between v/c ratio and fatal accident frequency. Initially, as traffic density increases but a high rate of speed is still maintained, the number of fatal accidents rises at an increasing rate. As density increases further, speed is compromised and fatal accidents increase at a decreasing rate, before decreasing as congestion continues to reduce speeds [17]. While results from Frantzeskakis and Iordanis [14] do not appear to support this hypothesis, the small dip in accident rates at high traffic volumes observed by Raff [8], as well as the curve found by Veh [7] give plausibility to the idea.

Models relating fatal accidents to traffic density and speed [29] supported the hypothesis of Shefer [17]; however, data relating accident rates to speeds was not available so the model was tested using simulated data. Also testing this hypothesis, time of day was used as a proxy for congestion. Data from the US, Israel, and Germany was analyzed and declines in fatal accidents were observed during the morning peak hours, assumed to be between 7 and 9 am [29]. This was thought to be a result of congestion at these times [29]. However, the morning peak hours are simply assumptions of periods of peak congestion—accident rates were not related to actual congestion at the time of the accident. The analysis of the results from these three countries also failed to identify any significant declines in accidents during the typical evening peak hours, which was attributed to a larger variation in work end times compared to start times [29]. The use of a simulated dataset and time of day as a proxy for congested periods limits the viability of these results. The importance of using direct congestion data related to the occurrence of traffic accidents is also highlighted in research conducted in London, where inner and outer London were used as a proxy for levels of congestion [48]. The results of the study were inconclusive, possibly because the spatial proxy for congestion is weak—congestion can be highly localized [49] and categorizing a large area as congested is not necessarily accurate.

In France, traffic volume data was collected every 6 mins in segments along 2000 km of highways, producing 2.9 million section-hour data points [16]. It was found that accident rates were highest when traffic volume was under 400 vehicles per hour and lowest at flows between 1000 and 1500 vehicles per hour [16]. As traffic volume increased above 1500 vehicles per hour the accident rate also increased, again displaying the characteristic U-shaped function. The high temporal resolution of the traffic data used in this study provides credibility to the viability of the U-shaped function between the two variables. However, while it may be logical that using higher resolution data could serve to uncover this second order polynomial term, it may be possible that the use of data taken from a heterogeneous range of study locations would hide more detailed relationships, resulting in the simple linear correlation which is often found [50].

When using aggregated data from four classes of roads, Dickerson, Peirson and Vickerman [50] produced the typical linear relationship between traffic flow and number of accidents. However, when the data was disaggregated into A, B, C and unclassified roads in inner and outer London, it was found that there was generally an increase accident rates in the upper range of traffic flows. This brings up an important point for the use of spatially expansive data sets. Analyzing a heterogeneous range of roads as one unit may sacrifice the detail of results on a smaller scale or for particular road types; instead, providing an averaged relationship for the area as a whole. Results obtained from aggregated data also have limited application outside of the study area—using disaggregated data allows knowledge gained to be applied to other settings [51]. Sullivan [51] used both aggregated and disaggregated data from Californian freeways. A simple positive correlation between accidents and hourly traffic volumes and the percentage of commuting periods where congestion was present was found when using the aggregated data, but the specific effects of queuing could not be determined. By disaggregating the data into queued and non-queued conditions, it was uncovered that accident rates were around two times higher in queued conditions for injury/fatal and non-injury accident types. This is an example of the ability of data disaggregation to uncover further detail between accident occurrences and causative factors. Jara-Díaz and González [52] determined that the effects of ADT on accident occurrence were greatly underestimated when fitted to a linear model. When a quadratic term was used, the influence of the traffic volume variable increased by around 40% and the effect of road geometry variables were more strongly captured. This suggests that the ability of high-quality data to unveil any quadratic relationships may allow more in depth modelling of the effects that influence accident occurrence [52].

Following a wide range of studies which use v/c ratios as a measure of congestion, Wang (2009) employed an equation for a ‘congestion index’, after suggesting that v/c is a proxy for congestion and the two are not equivalent. This equation, as used by Taylor, et al., [53], takes the difference between the time taken to travel a segment and the free flow travel time of that segment and divides it by the free flow travel time. The resulting value is independent of road segment length and road geometry, allowing it to be compared between different road segments [49]. This is different to the congestion index used by Woo [10], which was effectively a v/c ratio.

This index was used to investigate the effects of congestion on accidents on the M25 motorway in London, but it was found to have no impact on the frequency of accident occurrences [49]. Wang, Quddus and Ison [6] included major roads surrounding the M25 in the investigation, providing greater spatio-temporal variations in the occurrence of traffic accidents and levels of congestion. After this modification, increased congestion was found to result in increased fatal or serious injury accidents; possibly due to speeds remaining relatively high on major roads—even in congested conditions. Wang, Quddus and Ison [6] also used a model to predict traffic accidents using traffic delay, and presented a function similar to the hypothesis proposed by Shefer [17]. Theoretically, this means that in certain very high congestion scenarios, a reduction in congestion could actually lead to an increase in traffic accidents as conditions move back up towards the peak of the curve [6]. Although, these high levels of congestion were very rare, with only 0.8% of accident observations by Wang, Quddus and Ison [6] occurring above the level which relates to peak predicted accident rates. This may explain the simple positive correlations seen between annual average daily traffic and accident rates in a number of studies; the traffic volume may not have reached levels high enough for a decline in accidents to occur.

The same congestion index used by Taylor, Woolley and Zito [53]; Wang, Quddus and Ison [49]; and Wang, Quddus and Ison [6] was employed by Sun, et al., [54] in Shanghai, China, where the congestion index had a significant positive effect on the occurrence of accidents on urban expressways.

While older studies often focused on the effects of only a few variables on accident likelihood, newer research often considers larger numbers of factors. Such as Abdel-Aty and Radwan [55], who fit a negative binomial model to variables including AADT and roadway characteristics such as degree of curve and shoulder, median, and lane width. Increased AADT was found to result in increased frequencies of accidents and was the most critical of all factors investigated. In intersections in China, ADT, ratios of turn lanes, and average speed all positively affected accident rates. The distance between intersections was negatively correlated and intersections on one-way roads were found to have 47% less crashes than two-way roads [56,57]. Interestingly, intersections which were below elevated roads also experienced increased accident rates [56,57].

## 4. Applications

The ability to monitor traffic conditions in real-time and act to reduce accident prone conditions before they can fully develop is the ultimate goal, with models being critical tools to obtain a predictive understanding of accident risk. A comprehensive review of real-time crash prediction models has recently been presented by Hossain, Abdel-Aty, Quddus, Muromachi and Sadeek [19]. Most models reviewed use empirical, multidimensional methods with different approaches of factor pre-selection. Models showed a dominance of Bayesian approaches, suggesting an importance of using informative priors in these models [19]. Performance increases in models are thus based on our understanding of causative factors. Madanat and Liu [18] proposed a system for providing real-time predictions of the likelihood of incidents, where surveillance sensors would be used to measure traffic and environmental variables. If the combination of real-time conditions led to a high likelihood of incidents then traffic could be managed to reduce the likelihood of accident occurrence [18]. Oh, et al., [58] took this concept and applied it to a freeway in California where traffic data was collected every ten seconds using double inductive loop detectors and paired to 52 accidents. It was determined that the standard deviation of speed was the best indicator to identify the difference between disruptive (potentially leading to an accident) and normal (not leading to an accident) traffic conditions. More indicators, such as congestion or weather could be used in theory, however the small dataset of only 52 accidents did not allow for statistically significant multivariate analysis, and the data must be available in real time to be able to predict increased accident likelihood [58]. Real-time data for the standard deviation of speed was collected every ten seconds and averaged over five-minute periods. If the likelihood of accident occurrence increased then measures such as changing electronically controlled road signs [58,59], broadcasting advisory radio messages, or displaying information on in-vehicle navigation systems [59] could be taken to alleviate the dangerous conditions. These approaches could be categorized as components of freeway management systems, which Olmstead [60] found to reduce the frequency of property damage, possible injury and minor injury accidents, as well as side-swipe and rear end collisions.

However, the use of inductive loop detectors in traffic measurement may be unfavorable due to their high failure rates and the difficulty of cutting into the ground and stopping traffic to perform maintenance [61]. Ahmed and Abdel-Aty [61] suggest the use of existing AVI systems, which read motorists toll road tags as they pass through toll gates, to measure traffic conditions. Similar to Oh, Oh, Ritchie and Chang [58] the log of the coefficient of the variance of speed was found to be significant in predicting accident prone conditions [61]. In Colorado, AVI sensors were paired with RTMS and real-time weather information [62]. The model created with this combination of data sources achieved an 89% success rate when identifying crash cases in the dataset.

Urban arterials were investigated in Greece by Theofilatos [63], where traffic variation was found to significantly influence accident likelihood, and Theofilatos, et al., [64], where increased occupancy and the transition from low to high occupancy conditions led to increased accident likelihood.

### Bluetooth Traffic Measurement

Of particular interest for use in real-time accident prediction models, or even simple univariate analysis, is road segment travel time data collected from Bluetooth sensors. The large proportion of accident management studies which have been discussed in in this review focus solely on freeways [23], leaving a gap in urban research. While AVI and RTMS data is usually available on freeways, inductive loop detectors and Bluetooth can be used on urban arterials [20]. Bluetooth detectors are a type of wireless probing technology, and detect vehicles which contain a Bluetooth device in ‘discoverable mode’ [20]. The unique media access control (MAC) address of each device is logged at two points along a segment—travel time and average speed of the vehicle is then calculated based on this [20]. Wireless probe techniques for traffic travel time measurement can work by either logging vehicle positions at two fixed points at either end of a segment, as in AVI and Bluetooth based systems; or by randomly sampling wireless device locations over a network [65]. While the latter method does not require the infrastructure of fixed location measurement points, it is limited by reduced location accuracy [65].

One of the first ventures into the use of MAC address “sniffing” for the application of travel time measurement was undertaken by Wasson, et al., [66] in Indianapolis. During initial testing only around 1% of the ADT was recorded, which can be partially attributed to MAC addresses only being recorded the first time they passed through the segment, rather than each time as would be the case in proper deployment of the technique. For example, while still a relatively low proportion, Haseman, et al., [67] found that around 8% of passing vehicles were detected. The technology was also deemed to be one of the only ways to easily measure travel times in construction zones, due to the difficulty of reliably estimating road capacities [67]. Additionally, this new technology may present a much more cost effective solution to traffic measurement [67] than more traditional methods such as license plate recognition [68], GPS [69,70], or odometer reading from technicians’ cars to record travel times across segments [66]. Despite the benefits of wireless probe methods of vehicle tracking there are issues. Firstly, the accuracy of MAC address tracking through Bluetooth can introduce spatial error, although over the length of sufficiently long Bluetooth segments these errors are less of an issue [66]. Secondly, the temporary deviation of probed vehicles from the segment to make short stops, before continuing though the second Bluetooth detector, can incorrectly inflate travel time measurements [66]. This problem is more pertinent on urban arterials, which have more stopping locations [66]. Pedestrians and people riding busses who are using Bluetooth devices are also an issue for this technology, as their travel times through the Bluetooth segments will not necessarily be representative of the travel time taken for vehicles in the main traffic stream [65].

Kitali, et al., [71] used Bluetooth sensors to estimate the likelihood of secondary crashes occurring as a result of an initial incident. Traffic speeds were collected with MAC address logging to identify when traffic conditions were affected by the occurrence of an incident, allowing the frequency of secondary crashes to be determined.

In combination with traffic signal phase, traffic volume, and weather data, Bluetooth sensors were used on urban arterials and intersections in Florida in modelling the influence of these factors on the occurrence of accidents [20,21,22]. Average speed, left-turn volume, downstream green ratio, and rain were found to be significant indicators of accident risk on arterials [20]; and speed, among 14 other variables, was significant in predicting crashes in intersections [22]. While traffic and traffic signal data for five minute intervals before each crash were used in this study, it is suggested that higher temporal resolution one-minute data could be used in the future to relate accidents to the conditions at the exact time of the accident [20]. However, Roger and William [72] believe that one and five minute intervals of traffic volumes may be statistically unstable.

In recent studies, Bluetooth travel time measurements are generally used in combination with a range of other real-time measurements to model the influencing factors of traffic accident frequencies. While this is necessary for novel traffic management systems which use a range of real-time data to predict accident risk in real-time, the added complexity of such techniques may limit traffic studies in areas where this data is not available. In China, for example, Xie, Wang, Huang and Chen [57] mentions studies being limited to simple linear regression analysis due to limited crash and traffic operation data. Going back to traditional studies, which often used highly averaged measures such as AADT and ADT, could high temporal resolution Bluetooth data substitute these poorer quality data sources? This would allow much simpler analyses that could look at the basic relationship between accident frequency and congestion in areas where multiple real-time data sources are not available. While MAC address sensing records travel times along segments, delay can be calculated based on the difference to free-flow travel time; and statistics such as excess delay can be calculated by comparing delay to the usual delay at that time and day of the week. These numbers could then be used as a measure of congestion. Although, oversimplifying the model by only considering congestion could result in an “omitted-variables bias” [73]. Using data from crash reports for variables such as whether conditions and light conditions could reduce this bias by enabling more variables to be included without the need for multiple real-time datasets.

## 5. Discussion

Throughout the course of research into the effects of congestion on the occurrence of traffic accidents, the datasets used have changed considerably. Earlier studies tend to use highly averaged data such as AADT or ADT, with trends towards higher temporal resolution hourly data being seen in more modern research. While Gwynn [12] suggested that hourly traffic data would provide a stronger relationship between accidents and traffic volume, a better understanding of how the characteristics of data used affects the results achieved would drive future research choices. Table 1 presents a range of studies discussed throughout this review; summarizing the characteristics of the traffic data used, and the relationship discovered between accident occurrence and traffic conditions. The number of traffic data points used in each study is also included in the table to gauge the effect of dataset size on the ability to uncover a relationship.

Table 1 provides an overview of the results of the studies discussed throughout this review in combination with the characteristics of the traffic data used. This table only includes studies where spatio-temporal details of the data could be determined. Studies using models which assumed linearity (i.e., they can’t conclusively say if the relationship was linear or if there was a significant second order term) were excluded.

Both publication [16] and [50] found concave relationships between traffic volume and accident occurrence, possibly at least partially as a result of the large extent of the traffic volume datasets used. Both studies used hourly traffic data, with Martin [16] having 17,520 hourly traffic measurements (2 years × 365 days × 24 h) per measurement location and Dickerson, Peirson and Vickerman [50] having 21,900 (2.5 years × 365 days × 24 h). Gwynn [12] also reported a concave relationship while using a large traffic volumes dataset. Zhou and Sisiopiku [15] found a concave relationship while only using 768 data points, but the significance of the second order term in the regression was not reported.

In comparison, the studies which showed linear results more often used much fewer data points (Table 1). This may support the theory that hourly data shows a stronger relationship to accident occurrences [12]. However, studies [9] and [54] produced linear relationships despite having many traffic volume data points (Table 1). This could be explained by their use of ADT, which averages variations in traffic volume throughout the day, limiting the detail which can be found between traffic volumes and accident occurrences.

Cadar, Boitor and Dumitrescu [38] argued a convex relationship, however the use of only seven data points and the lack of statistical comparison assessing superiority of the nonlinear over a linear model make these results questionable. Wang, Quddus and Ison [6] report convex response of accidents to total yearly traffic delay, but the second order term was not statistically significant.

### Future Research Direction

Based on the information presented in Table 1, future directions for research, particularly relating to data collection, can be suggested. Traffic datasets with large numbers of traffic volume measurements appear to be beneficial in uncovering significant second order terms, as are high temporal resolution measurements, with Schoppert [9] and Sun, Li, Li and Chen [54] finding the simple linear correlation when using ADT, despite having large numbers of traffic measurements. Future studies will benefit from using large numbers of high temporal resolution (hourly or less) traffic volume measurements.

Based on the limited number of studies using Bluetooth detection for traffic measurement, the continued development and use of this technique in accident analysis would be interesting. Bluetooth MAC address probing techniques are also able to collect high temporal resolution data by measuring travel times of each Bluetooth enabled device to pass along a segment, which the results in Table 1 suggest to be beneficial in uncovering detailed results.

Focus in future research could lie in the simplification of models used to analyze traffic accident frequencies. While many current studies focus on modelling the effects of a range of factors on accidents, applying Bluetooth data in simplified studies would still be of use. As suggested by Mannering and Bhat [74], simplified models may be necessary for situations where the data needed for complex multi-variate models is not available. High temporal resolution Bluetooth data could effectively replace the highly aggregated traffic volume measures used in traditional studies to potentially see beyond the basic linear relationship. Most studies reviewed by Hossain, Abdel-Aty, Quddus, Muromachi and Sadeek [19] were using sample sizes of accidents paired with detector data below 500; the continued use of modern approaches such as Bluetooth measurement would allow sample sizes to be increased by several orders of magnitude by taking measurements at much finer temporal scales.

Future studies should also use disaggregated data to avoid any relationships being hidden when data from a heterogeneous study area is averaged into a single analysis, based on results from Dickerson, Peirson and Vickerman [50] and Sullivan [51].

## 6. Conclusions

This review has demonstrated that our understanding of how traffic congestion affects accident risks is still limited. The literature generally shows an increase in accidents with increasing levels of congestion/traffic volume. Highly averaged measures of traffic volume such as AADT and ADT, however, can limit how detailed of a relationship can be observed between accident rates and congestion. Finer temporal resolution traffic data furthermore allows more factors to be considered as dependent variables and a more complete picture of the causative variables for accident frequency to be built.

The importance of mitigating congestion in reducing delays and the resulting loss of economic productivity [37], amongst an array of other benefits, highlights the need to understand the effects of congestion on traffic accidents—how would reducing congestion for these reasons change the frequency and severity of accidents? While many studies have looked at this relationship there is still a strong debate. This lack of consensus may come from subtle differences in characteristics between study locations and the large number of covariates, but also from limitations in the design and narrow focus of past research. For example, many studies only focus on highway segments, where results may not be applicable in all situations due to highways being relatively free flowing, even in high traffic volume conditions [6], unlike urban roads. The characteristics of the data used also appear to affect the results which different studies presented. When using AADT results often pointed to a simple positive correlation, with more detailed relationships being uncovered with higher temporal resolution data. Therefore, it could be concluded that if further research was to be conducted it would be preferable for the datasets used to have a spatial range including both freeway and urban areas. High resolution spatio-temporal traffic monitoring techniques such as Bluetooth MAC address sensing is a novel technique which could be used to collect this high-quality traffic data. The majority of previous studies using this technology include a range of other variables in their modelling, such as real-time weather and traffic signal data. On the basis that high resolution traffic data can uncover more detailed relationships to traffic accident occurrence, it is possible that travel time data from Bluetooth sensors could be used in place of highly averaged measures of traffic volume. This could then be used in more simple modelling techniques for use by local councils where the lack of large amounts of real time data do not permit the complex modelling which is at the current forefront of real-time traffic management research.

The need for this improved study design is necessary to take full advantage of advanced traffic measurement systems which are capable of recording traffic characteristics in real-time. Having access to spatially explicit, high temporal resolution, real-time traffic data can allow undesirable conditions to be avoided before they can fully develop [58]. However, the lack of understanding of the causes of traffic accidents limits the use of these systems in identifying conditions which would be considered accident prone. This highlights the necessity of more long term, broad scale studies, using advanced traffic data to continue to understand the uncertainties which remain in the wake of research into the topic thus far.

## Figures and Tables

**Table 1:** Summary of relationships between congestion and traffic accident frequency and the spatio-temporal resolution of the traffic data used. “Concave” relationships encompass the U-shaped relationship mentioned throughout the review as well as any positive second order polynomial terms in linear models. Inversely, “convex” relationships include the inverse U-shape as well as any negative second order terms. The number of data points were calculated by the multiplication of the “number of units” and “number of measurement locations” columns, if not explicitly stated in the publications.

Relationship	Temporal Unit	Duration	Number of Units	Number of Measurement Locations	Number of Data Points	Publication
Linear	Year	1954–1955	2	987	1974	[10]
Linear	Year	1985	1	399	399	[39]
Linear	Year	1954–1955	2	426	852	[11]
Linear	Day	1955	365	1374	501,510	[9]
Linear	Day	2010–2013	1095	167	182,865	[54]
Concave	Hour	1997–1998	17,520	92 (×2 directions)	2,900,000 (after filtering)	[16]
Concave	Hour	1959–1963	1825	1	43,800	[12]
Concave	Hour	1993–1995	21,900	54 (×2 directions)	2,365,200	[50]
Concave	Hour	1993–1994	768 (for weekdays)	1	768	[15]
Convex	Year	2015	1	7	7	[38]
Convex	Year	2003–2007	-	-	1391	[6]
